# Harnessing plant biosynthesis for the development of next-generation therapeutics

**DOI:** 10.1371/journal.pbio.3002886

**Published:** 2024-11-05

**Authors:** Philip Spence, James Reed, Anne Osbourn

**Affiliations:** Department of Biochemistry and Metabolism, John Innes Centre, Norwich Research Park, Norwich, United Kingdom

## Abstract

Genomics-based predictions indicate that plants harbor the ability to make a vast array of as yet undiscovered chemistry. This Perspective argues that recent advances open up the potential to harness this capability at unprecedented scale for the discovery and development of new drugs.

## Teaser

Genomics-based predictions indicate that plants harbor the ability to make a vast array of as yet undiscovered chemistry. Recent advances open up the potential to harness this capability at unprecedented scale for the discovery and development of new therapeutics.

Although the number of chemically synthesizable compounds has increased over the last 30 years, this has not led to an increase in drug approvals [1]. It is speculated that the quality of the chemical structures, rather than the quantity of new molecules, is the limiting factor, i.e., it is the restricted “chemical space” accessible via synthetic chemistry that is holding back the success rates in drug discovery and development. This has led to the conclusion that, in order to discover the next generation of drugs, it is necessary to expand the accessible chemical space that is being searched.

Plants have been used by humans for thousands of years as a source of traditional medicines and drugs. The handful of well-known drugs currently sourced from plants (e.g., morphine, paclitaxol, galanthamine, and artemisinin) represent only the tip of the iceberg. Predictions based on genomic studies suggest that the Plant Kingdom collectively harbors the ability to make millions of structurally diverse molecules, the vast majority of which have not yet been described. Because of their structural complexity, these molecules are for the most part beyond the reach of chemical synthesis, at least at commercial scale.

Natural products are biosynthesized by living organisms through sequences of multi-step enzymatic transformations to yield molecules that are often structurally elaborate. These molecules have been honed by evolution to interact with biological targets and modulate biological processes and so are a rich source of bioactive molecules. By learning how plants make chemicals and isolating the genes responsible, it becomes possible to harness this capability to biosynthesize natural products and analogues in heterologous expression systems, opening up new chemical space for drug discovery. This strategy has been widely exploited in microbes since the early 2000s, when the advent of genome sequencing opened up opportunities to discover biosynthetic pathways for antibiotics and other small molecules using genome mining-based approaches [2]. Although plants have a long history of medicinal use, they are largely untapped in terms of their potential for drug discovery, and have lagged behind microbes because of their large and complex genomes [3], as well as the challenges of expression and reconstitution of plant biosynthetic genes and pathways in heterologous systems (traditionally microbial ones, such as *Escherichia coli* and bakers’ yeast) [4].

Over 200,000 natural products have been reported from plants to date [5], but the biosynthetic pathways for most of these are unknown. Furthermore, it is clear that the vast majority of the metabolic diversity represented within the Plant Kingdom awaits discovery, since genome sequencing has revealed that around 20% of the genes within plant genomes have predicted functions in specialized metabolism [6]. There are approximately 500,000 species of higher plants on Earth.

Genome sequences are currently available for around 1,500 of these, but that figure is set to explode with the onset of large-scale genome sequencing projects (such as the Earth BioGenome Project) [3]. This burgeoning body of plant genome sequence data harbors the instruction manual needed to make the chemical diversity produced by the Plant Kingdom. The development of powerful new computational strategies now makes it possible to rapidly scavenge through plant genome sequences to identify candidate genes for new enzymes, pathways, and chemistries [7]. Inventories of triaged gene sets can then be prioritized and cloned ready for functional analysis. The bottlenecks associated with expression of plant biosynthetic enzymes and pathways in microbial systems can be circumvented by instead using transient plant expression technology [8,9], which offers a rapid and easy means of testing the functions of single genes and combinations of genes, so enabling the de novo elucidation of complex multi-step biosynthetic pathways (see below).

Although microbial systems such as yeast tend to be the default systems for heterologous expression, certainly by industry, microbes lack many of the features needed for efficient and effective expression of plant natural product biosynthetic enzymes and pathways [4]. Transient plant expression technology offers a solution to this problem [8,9]. This approach (also known as agro-infiltration) takes advantage of the natural ability of the bacterium *Agrobacterium tumefaciens* to transfer genetic material inside a plant cell. *A*. *tumefaciens* cells harboring expression constructs for one or more genes of interest are infiltrated into the leaf of a suitable host—usually *Nicotiana benthamiana*, a wild relative of tobacco, which is particularly amenable to this process. Upon transfer of the target gene(s) into the plant cell, the enzyme(s) encoded by this transferred DNA are expressed and their functions can be evaluated. A key advantage of this approach is that it is extremely rapid—the time from agro-infiltration to analysis of the metabolite content of leaf extracts takes just 3 to 5 days [9]. A growing number of increasingly complex plant natural product pathways have been successfully elucidated by transient expression in *N*. *benthamiana* and the pathways reconstituted in this heterologous host [10,11], including our recent demonstration for the 20-step pathway for the biosynthesis of the vaccine adjuvant QS-21, normally sourced by extraction from the bark of the Chilean soapbark tree *Quillaja saponaria* [12].

As well as being used for reconstitution of pathways for specific compounds, transient plant expression can also be deployed to mix and match enzymes from different plant species.

Because higher plants have evolved relatively recently in evolutionary time, their biosynthetic enzymes are for the most part interchangeable. This enables toolkits of hundreds of enzymes for the biosynthesis and decoration of pharmaceutically important scaffolds from diverse plant species to be deployed using combinatorial biosynthesis, so enabling systematic investigations of the structure-activity properties of suites of structurally related molecules (e.g., [9]).

Agro-infiltration of *N*. *benthamiana* is usually carried out manually by pushing the bacterial suspension into the leaves using a syringe without a needle for small-scale analytical work. However, where larger quantities of product are needed for purification and structural validation by nuclear magnetic resonance (NMR) and for evaluation of bioactivity, the process can be linearly and reliably scaled simply by increasing the numbers of plants and introducing the *Agrobacterium* cells into whole plants by vacuum infiltration [9]. Indeed, this has already been done on an industrial scale for the production of proteins and there are regulatory-compliant facilities in North America that have been purpose-built for this reason. Even at laboratory scale, vacuum infiltration has been used to produce gram-scale amounts of product, way beyond the milligram amounts normally required for most bioassays [9]. This paves the way for taking prioritized lead compounds with promising therapeutic properties forward for early stage evaluation and optimization, with the potential to scale up further as needed.

**Fig 1:**
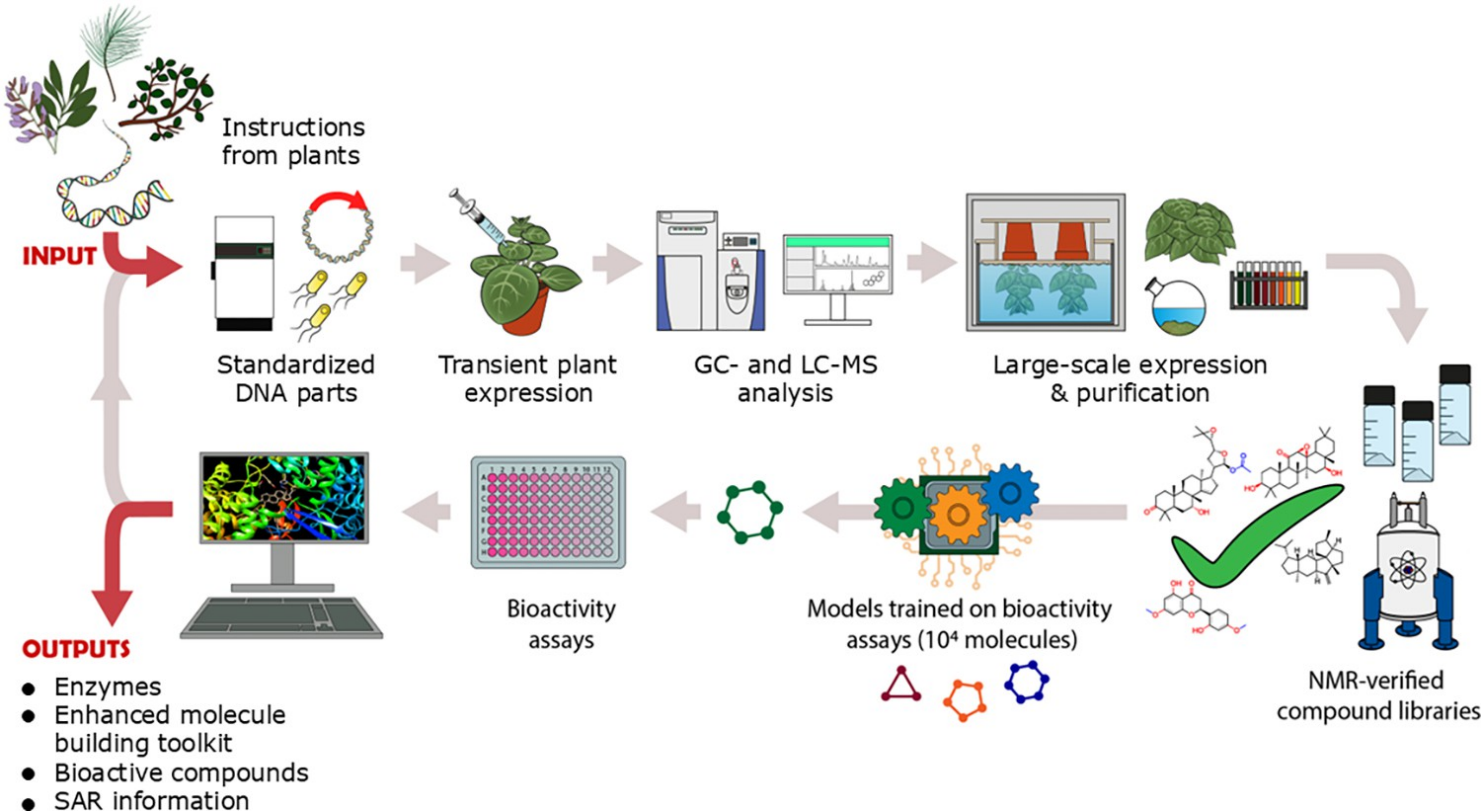
Engineering biology to harness the potential of plants in the production of next-generation therapeutics. Transient plant expression provides a rapid means of generating target molecules at preparative scale for evaluation of their pharmacological properties. Lead molecules can then be further optimized in an iterative design-build-test-learn cycle, so providing an entirely new pipeline for next-generation drug discovery. Machine learning (ML) models trained on bioactivity data can be integrated into this pipeline to triage virtual compound libraries and target molecules for biosynthesis based on activity predictions.

The use of plants as sources of, or inspiration for, new drugs has traditionally been hindered by several factors: (i) screening of plant extracts for bioactivity may lead to hits but it may not be possible to isolate a single bioactive determinant; (ii) the target compound may be present in plant extracts only at very low levels, as part of a complex mixture; (iii) the producing plant may be a wild (even an endangered) species that only grows in a particular part of the world, rendering commercial-scale extraction unviable; and (iv) the target compound may have promising bioactivity but need optimization, which is made difficult by the recalcitrance of most natural products to chemical synthesis and precisely directed modification. The key computational and technological advances outlined above now make it timely to take a different more systematic approach to unlocking and harnessing the chemical engineering capabilities of the Plant Kingdom (Fig 1). The DNA-encoded instruction manual harbored within plant genomes can now be deployed to make and optimize a swathe of new chemistry. Ultimately, this will enable the collective chemical engineering capabilities of the Plant Kingdom to be garnered in order to access and engineer previously inaccessible chemical space by directed biosynthesis, to deliver a new “golden age” of natural product drug discovery and development.
